# Macrophage Migration Inhibitory Factor Interacting with Th17 Cells May Be Involved in the Pathogenesis of Autoimmune Damage in Hashimoto's Thyroiditis

**DOI:** 10.1155/2015/621072

**Published:** 2015-03-15

**Authors:** Haibo Xue, Yuhua Yang, Ying Zhang, Shoujun Song, Li Zhang, Lei Ma, Tingting Yang, Huan Liu

**Affiliations:** ^1^Department of Endocrinology and Metabolism, Binzhou Medical University Hospital, 661 Second Huanghe Road, Binzhou 256603, China; ^2^Department of Endocrinology, Wudi County People's Hospital, Tengda Street, Wudi 251900, China; ^3^Department of Clinical Nutrition, Binzhou Medical University Hospital, 661 Second Huanghe Road, Binzhou 256603, China; ^4^Department of Dermatology, Binzhou Medical University Hospital, 661 Second Huanghe Road, Binzhou 256603, China

## Abstract

*Purpose*. To explore the possible role of MIF and Th17 cells in the thyroid-specific autoimmune damage of Hashimoto's thyroiditis (HT). *Material and Methods*. We enrolled 40 HT patients and 30 healthy controls and divided HT patients into euthyroid subset (*n* = 22) and subclinical or overt hypothyroidism subset (*n* = 18). The percentages of Th17 cells and expressions of MIF, interleukin 17A (IL-17A) mRNA in PBMCs, as well as serum concentrations of MIF, and IL-17A, and thyroid functions, and thyroid-specific autoantibodies (TPOAb, TgAb) were detected by flow cytometry, real-time RT-PCR, ELISA, and ECLIA in all subjects. *Results*. MIF mRNA, IL-17A mRNA expressions and Th17 cells percentages, serum MIF, and IL-17A protein levels were all significantly higher in HT patients, even in euthyroid subgroup. Additionally, the differences became more obvious in dysfunction subgroup. Importantly, both MIF levels and Th17 cells percentage were positively correlated with serum TPOAb, TgAb, and thyrotropin (TSH) levels in HT patients. *Conclusions*. These data suggest that MIF and Th17 cells increased dynamically and positively correlated with the markers of thyroid autoimmune damage, which indicated that interaction between MIF and Th17 cells may participate in the pathogenesis and development of thyroid-specific autoimmunity in HT.

## 1. Introduction

Hashimoto's thyroiditis (HT) is known as a typical autoimmune thyroid disease (AITD), which affects up to 2% of general population and is 5 to 10 times more common in women than in men [1, 2]. However, the prevalence of anti-thyroid antibodies without any clinical manifestation may be even higher [3, 4]. The features of HT mainly include lymphocytic infiltration in the thyroid and increasing serum antibodies to thyroid-specific antigens (thyroid peroxidase antibody, TPOAb; thyroglobulin antibody, TgAb). Most of HT patients ultimately evolve into hypothyroidism, although patients can have normal thyroid function or even hyperthyroidism at early disease stage. Therefore, it has been considered the most common cause which leads to hypothyroidism [5, 6]. The crucial factor in the development of HT is considered the breakdown of immune tolerance. Furthermore, hereditary susceptibility and environmental factors may increase the incidence of HT, especially high iodine intake [7]. So far, the exact pathogenesis of HT has not been well elucidated.

Macrophage migration inhibitory factor (MIF) is originally discovered as a lymphokine relevant to delayed-type hypersensitivity, which inhibits the random migration of macrophages and concentrates macrophages at inflammatory lesions as well [8]. As a multifunctional proinflammatory cytokine, MIF is demonstrated to participate in innate and adaptive immune responses and is known to be implicated in the pathogenesis of many autoimmune diseases, such as inflammatory bowel disease (IBD) [9], rheumatoid arthritis [10], and vitiligo vulgaris [11]. Additionally, MIF gene polymorphisms (rs755622 SNP) have been shown in association with the severity of goiter in patients with untreated Graves' disease (GD), which is another common AITD [12]. More recently, T helper 17 cells (Th17), a newly recognized subset of CD4^+^ T helper cells, have been demonstrated to play critical roles in the pathogenesis of several autoimmune diseases, which is considered the main source of interleukin 17 (IL-17) [13–15]. Given that subsequent investigations reported that MIF can interact with other cytokines and lead to impaired immune responses, there is no in-depth study about the role of MIF in HT. Several studies have revealed that MIF is involved in promoting the differentiation and development of IL-17 in animal [16, 17]. Thus we speculate that MIF may be implicated in inflammatory and autoimmune disease through interacting with Th17 cells.

Therefore, in the present study, we detected the expressions of MIF, Th17 cells, and IL-17A in HT patients and healthy controls, analyzed the relationships between MIF and Th17 cells and MIF, Th17 cells, and thyroid-specific autoantibodies, and tried to explore the possible role of MIF and Th17 cells in the pathogenesis of thyroid autoimmune damage in HT.

## 2. Materials and Methods

### 2.1. Subjects and Clinical Assessments

Forty patients with HT were enrolled in this study, and all the patients were newly diagnosed and untreated previously. The diagnosis of HT was based on the classical criterion [18]. HT patients were divided into two subgroups based on their thyroid functions: HT-A subset (euthyroidism, 22 cases) and HT-B subset (subclinical or overt hypothyroidism, 18 cases). Meanwhile thirty healthy volunteers with matched age and sex features were selected as the healthy controls (HC), who had no autoimmune disease history. The clinical features of all subjects were shown in Table 1. All study procedures were performed in accordance with the guidelines of the Declaration of Helsinki with the approval of the Ethics Committee of Binzhou Medical University Hospital. From all participants, a written informed consent was obtained.

Electrochemiluminescence immunoassay analyzer (ECLIA, Roche Cobas 6000, Germany) was used to measure thyroid function and thyroid-specific autoantibodies in all subjects, which include TSH, free triiodothyronine (FT_3_) and free thyroxine (FT_4_), and TPOAb and TgAb (their reference ranges were shown in Table 1). Color Doppler ultrasonography (LOGIQ9, GE USA) was performed on all subjects by trained observers, mainly observing thyroid size, echo (especially hypoechogenicity), and blood flow. Urinary iodine excretion was detected in all participants to evaluate their iodine nutritional status [19].

### 2.2. Flow Cytometric Analysis of Th17 Cells

Isolation of peripheral blood mononuclear cells (PBMCs) was performed via Ficoll-Hypaque density gradient centrifugation (Sigma-Aldrich, St. Louis, MO, 1200 rpm, 25 min). Cells were washed twice in phosphate buffered saline (PBS), incubated for 5 hours with 25 ng/mL phorbol myristate acetate (PMA) and 1 *μ*g/mL ionomycin (Sigma, USA) in the presence of 2 mmol/mL monensin at 37°C under a 5% CO_2_ environment, and then transferred to each tube, washed once with PBS, and incubated with anti-CD4-FITC at 4°C for 30 minutes in the dark. Following being fixed and permeabilized, cells were stained with intracellular anti-IL-17A-PE. Meanwhile isotype-matched controls were used to correct nonspecific binding. Th17 cell numbers were analyzed with a FACSCanto flow cytometer, and data were collected and analyzed using CellQuest software (BD Biosciences, USA). The antibodies mentioned above were obtained from eBioscience (USA).

### 2.3. Real-Time Quantitative RT-PCR Analysis of MIF and IL-17A mRNA

Total RNA was isolated from PBMCs with Trizol (Invitrogen, USA). Reverse transcription was performed with PrimeScript RT reagent kit (TaKaRa, Japan) on an ABI 9700 PCR meter (37°C for 15 min, followed by 85°C for 5 s). Real-time RT-PCR was performed on a Rotor-Gene 3000 (Corbett Research, Australia) using SYBR Premix Ex Taq II (TaKaRa, Japan) and the given primers for MIF and IL-17A (Table 2); additionally, *β*-actin was used as an internal control. The same sample was run in triplicate and data were analyzed with the Rotor-Gene Real-Time Analysis Software 6.0.

### 2.4. Measurements of Serum MIF and IL-17A Using Enzyme-Linked Immunosorbent Assay (ELISA)

The levels of serum MIF and IL-17A protein were measured in duplicate with human ELISA kits (R&D system, USA), and the protocols were conducted in accordance with the manufacturer's instructions.

## 3. Statistical Analysis

Data are shown as mean ± standard deviation or median (interquartile range) according to the distribution. The normally distributed data were analyzed by independent-samples *t*-test and Pearson correlation. Meanwhile, the analysis of abnormally distributed data was performed with Kruskal-Wallis test followed by the Mann-Whitney *U* test and Spearman test. All analyses were performed using GraphPad Prism software version 6.0 (San Diego, CA). *P* values of < 0.05 were considered statistically significant.

## 4. Results

### 4.1. Subject Characteristics

Thyroid function test revealed that TSH, FT_3_, and FT_4_ in HT and HT-B subgroup changed obviously compared to HC (all *P* < 0.05); however, the differences between HT-A subgroup and HC were not significant (all *P* > 0.05). Moreover, thyroid function in HT-B differed markedly from that in HT-A (*P* < 0.01 or <0.05). Additionally, serum TPOAb and TgAb titers increased significantly in HT and HT subgroups (all *P* < 0.01), and HT-B patients had higher levels than HT-A (*P* < 0.01). Moreover, the results of median of urine iodine (MUI) indicated that all participants were in adequate iodine status, and there were no significant differences between different groups (all *P* > 0.05). These data were shown in Table 1.

### 4.2. MIF mRNA and IL-17 mRNA Levels in PBMCs

**Figure 1:**
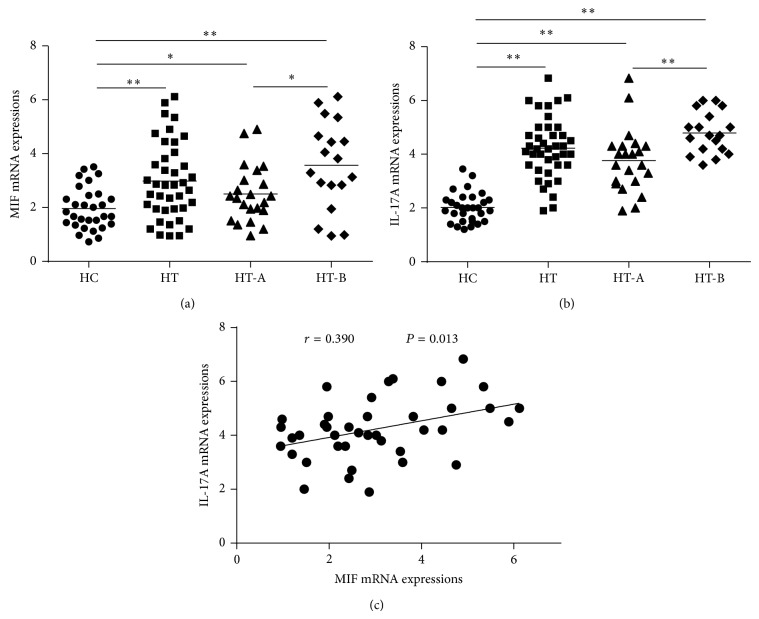
(a) Expressions of MIF mRNA increased significantly in HT patients compared with HC (^*^ *P* < 0.05, ^**^ *P* < 0.01). In HT subsets, HT-B patients had higher MIF mRNA expressions than those in HT-A subgroup (^*^ *P* < 0.05), which indicated that there were dynamic changes consistent with the severity of the disease. (b) The same characteristics of IL-17A mRNA in HT and HT subgroups as MIF mRNA expressions could be found (^**^ *P* < 0.01). (c) MIF mRNA levels positively correlated with IL-17A mRNA levels in HT patients.

The MIF mRNA expressions of PBMCs in HT (2.98 ± 1.42), HT-A (2.50 ± 1.04), and HT-B (3.57 ± 1.63) subsets increased significantly compared to HC (1.96 ± 0.78,  *P* = 0.001, 0.038, 0.001, resp.), and HT-B patients had higher levels than HT-A (*P* = 0.023), Figure 1(a). Additionally, the IL-17A mRNA changes of PBMCs in HT (4.23 ± 1.13), HT-A (3.77 ± 1.18), and HT-B (4.79 ± 0.77) were also much higher than HC (2.02 ± 0.56, all *P* < 0.01); moreover, the differences between HT-A and HT-B subgroups were significant (*P* = 0.003), Figure 1(b). Furthermore, Pearson correlation analysis showed that MIF mRNA levels positively correlated with IL-17A mRNA expressions (*r* = 0.390, *P* = 0.013), Figure 1(c). These data suggested that both MIF and LI-17A mRNA increased markedly in HT patients and had dynamic changes during different disease status. More interestingly, MIF mRNA expressions had a close relationship with that of IL-17A.

### 4.3. Percentage of Th17 Cells in PBMCs

**Figure 2:**
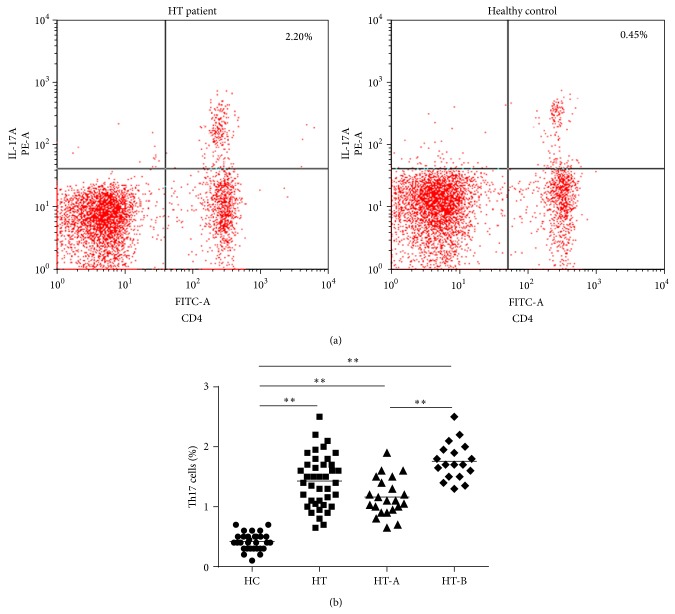
(a) The representative graphs of flow cytometric analysis. CD4^+^IL-17A^+^ T cells were determined as Th17 cells. (b) Comparisons of Th17 cells percentages between subjects in HT and HC, as well as between HT subsets and HC or between HT-A and HT-B subsets. HT patients had much higher Th17 cells proportion than HC, even in euthyroidism status (^**^ *P* < 0.01). Additionally, Th17 cells numbers in HT-B subgroup also increased significantly compared to HT-A (^**^ *P* < 0.01), which provided the evidences of dynamic changes in different disease stages.

Flow cytometry analysis showed that the proportions of circulating Th17 cells in HT (1.43 ± 0.43%), HT-A (1.16 ± 0.32%), and HT-B (1.76 ± 0.31%) were all obviously higher than healthy controls (0.42 ± 0.14%, all *P* < 0.01). In addition, HT-B subgroup patients also had higher Th17 cell numbers than HT-A (*P* < 0.01), Figure 2. These results revealed that peripheral Th17 cells dynamically changed between the two HT patient subsets, which suggested that there was a close relation between Th17 cells and the course of HT.

### 4.4. ELISA Results of MIF and IL-17A Concentrations in Serum

**Figure 3:**
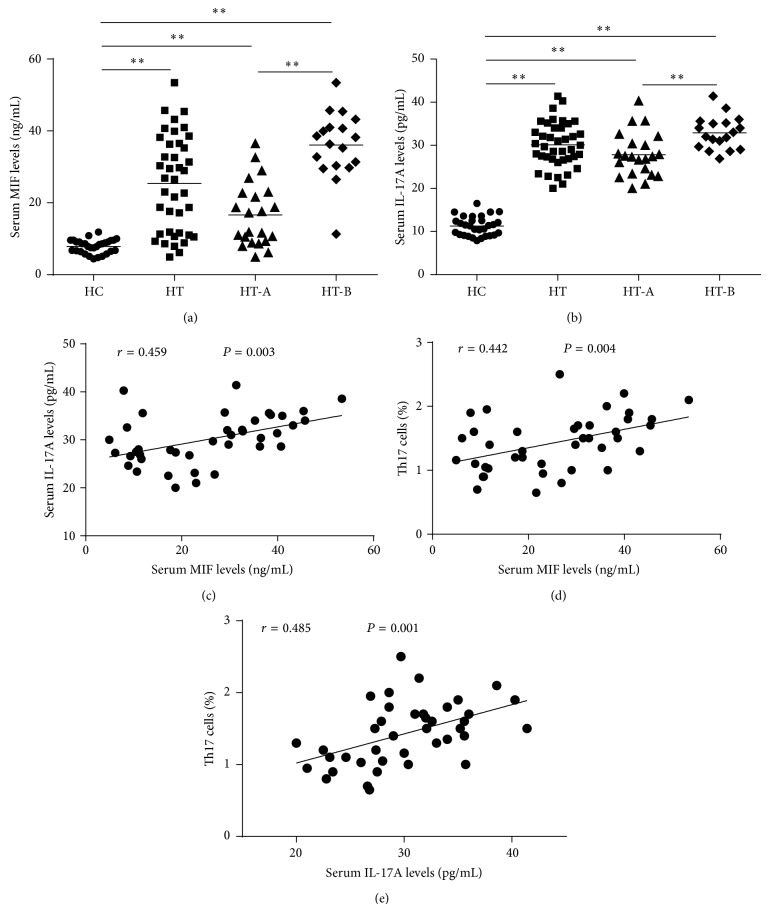
(a) Differences of serum MIF protein levels between HT and HC, HT subsets and HC, and HT-B and HT-A subgroups were all significant (^**^ *P* < 0.01). (b) Similar characteristics of serum IL-17A protein concentrations could be found like MIF (^**^ *P* < 0.01). (c) and (d) Positive correlations between MIF protein levels and IL-17A protein concentrations. Th17 cells percentages were found in patients with HT. (e) Levels of serum IL-17A protein positively correlated with the proportion of Th17 cells.

Increased serum MIF protein levels were found in both HT and HT subsets compared to HC (25.38 ± 13.27 ng/mL, 16.64 ± 8.93 ng/mL, and 36.08 ± 9.26 ng/mL* versus *7.89 ± 1.89 ng/mL, all *P* < 0.01); meanwhile, there were remarkable differences between HT-A and HT-B subgroups (*P* < 0.01), Figure 3(a). Additionally, serum IL-17A concentrations also increased obviously in HT and HT subsets patients compared to HC (30.09 ± 5.18 pg/mL, 27.80 ± 5.12 pg/mL, and 32.88 ± 3.76 pg/mL* versus *11.27 ± 2.23 pg/mL, all *P* < 0.01), and patients in HT-B subgroup had higher IL-17A levels than HT-A subgroup (*P* < 0.01), Figure 3(b). Furthermore, serum MIF protein levels positively correlated with serum IL-17A protein concentrations and Th17 cells percentages of PBMCs in patients with HT (*r* = 0.459, 0.442; *P* = 0.003, 0.004, resp., Figures 3(c), 3(d)). Similarly, a positive correlation was found between serum IL-17A levels and peripheral Th17 cells percentages in patients with HT (*r* = 0.485, *P* = 0.001).

### 4.5. Results of Correlation Analysis

**Figure 4:**
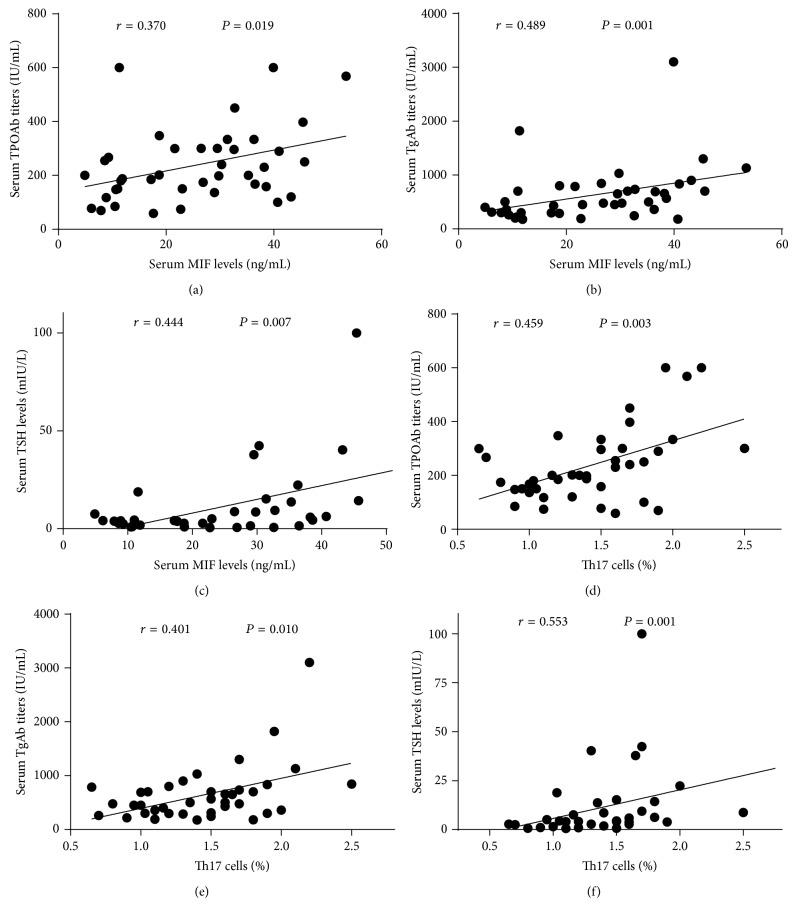
(a)–(c) MIF protein levels positively correlated with serum TPOAb, TgAb, and TSH concentrations. (d)–(f) Increasing percentages of Th17 cells also had positive correlation with serum TPOAb, TgAb, and TSH levels. These analyses all used Spearman test.

First, we analyzed the relationships between serum MIF levels and serum TPOAb and TgAb titers in HT patients. The results revealed that MIF had positive correlations with TPOAb (*r* = 0.37, *P* = 0.019) and TgAb (*r* = 0.489, *P* = 0.001). In addition, Th17 cells percentages also had positive correlations with TPOAb (*r* = 0.459, *P* = 0.003) and TgAb in HT patients (*r* = 0.401, *P* = 0.010). More interestingly, both MIF and Th17 cells positively correlated with serum TSH levels in patients with HT (*r* = 0.444, 0.553; *P* = 0.007, 0.001, resp.). These data suggested that there were close relationships between MIF and Th17 cells and the markers of thyroid-specific autoimmune damage, as well as the sensitive index of thyroid dysfunction, as shown in Figure 4.

## 5. Discussion

Although immunological factors have been considered to play crucial roles in the pathogenesis of AITD, the precise mechanisms by which immunological factors contribute to the pathogenesis of HT have remained obscure. In this work, we aimed to analyze the potential roles of MIF and Th17 cells in the thyroid-specific autoimmune activity in patients with HT. In accordance with what had been found in the patients with other autoimmune diseases, we also demonstrated increased MIF mRNA levels of PBMCs in HT patients. Meanwhile, serum MIF protein concentrations also increased, as well as positively correlating with MIF mRNA expressions. Moreover, dynamic alterations in different disease status were found to be statistically significant, even in euthyroid stage, particularly more obvious in patients with subclinical and overt hypothyroidism. Further detections illustrated that peripheral circulating Th17 cells and their main effective cytokine IL-17A increased markedly in HT patients with similar features as MIF. Meanwhile, both IL-17A mRNA in PBMCs and serum IL-17A protein correlated positively with Th17 cells percentages. Importantly, we further found that there were positive correlations between MIF and Th17 cells percentage, IL-17A levels in HT patients. Previous studies demonstrated MIF could induce powerful proinflammatory biological responses and has been shown to be an important effector molecule in infection [20] and also upregulate the expression of Toll-like receptor 4 (TLR4), which mediates lipopolysaccharide binding and activation of macrophages [21]. Therefore, MIF has been recognized as a cytokine that exhibits a broad range of immune and inflammatory activities, including induction of inflammatory cytokines, and regulation of macrophage and lymphocyte proliferation. MIF deficiency, whether achieved through genetic deletion (*MIF* ^−/−^) or anti-MIF antibodies (Abs) neutralization, results in inhibiting inflammatory responses in a variety of murine models of human inflammatory and autoimmune diseases, followed by significant reduction of inflammatory cell infiltration and cytokines expressions [22–24], whereas an increased number of skin-infiltrating eosinophils were observed in ovalbumin-sensitized MIF transgenic mice compared with the wild-type [25], further suggesting MIF plays a dominant role in lymphocyte activation and cytokine production. A recent study reported that MIF-knockout mice had severely impaired production of IL-17, IL-1*β*, IL-6, IL-23, and TGF-*β*, but treatment of lymph node cells with recombinant MIF upregulated antigen-stimulated IL-17 expression and secretion [16]. Because these cytokines are essential for the differentiation and sustained generation of IL-17 from naïve T lymphocytes [26, 27], thus MIF is considered to potently stimulate IL-17 production through a complex cytokine network. Th17 cells have been designated as predominant producers of IL-17 and been demonstrated to play a pivotal role in many kinds of autoimmune diseases [28], including some common AITD [29, 30]. Taken together with our results, MIF may be implicated in the pathogenesis and progression of HT through promoting the differentiation and development of Th17 cells.

In addition to lymphocytes infiltration, another important feature of HT is the increased levels of thyroid-specific autoantibodies, mainly including TPOAb and TgAb, which indicate the severities of autoimmune damage in thyroid [31]. In the present study, we firstly found that MIF mRNA and protein expressions positively correlated with TPOAb and TgAb titers in HT patients, further supporting a close relationship between MIF and thyroid autoimmune response. The current opinion is thyroperoxidase (TPO), relating to TPOAb, is the main disease-causing antigen (Ag) in human [32, 33]. TPOAb is found in about 95% of HT patients but is rare in healthy controls and correlates well with the number of autoreactive lymphocytes infiltrating the thyroid [34]. Therefore, it is now considered the best serological marker of thyroid dysfunction, and its presence is predictive of the subsequent occurrence of thyroid failure in AITD patients [6]. Importantly, another finding of our study is that MIF also positively correlated with serum TSH levels, which is the most sensitive index reflecting thyroid function. In addition, Th17 cells percentages in PBMCs had illustrated the same characteristics as MIF, positively correlating with TPOAb, TgAb, and TSH. It has been found that T cells activated by specific Ag, mitogens, or anti-CD3 Abs show increased expression of MIF mRNA and protein. However, anti-MIF Abs inhibit T cell proliferation and Abs production from B cells [35]. Thus, MIF expression has been considered to be critical to the generation of an antigen-specific immune response. Collectively, we consider that the severities of thyroid autoimmune damage and impaired thyroid function status may be attributed to the interaction between MIF and Th17 cells.

## 6. Conclusions

Taken together, our data provide novel evidence that increased MIF, together with circulating Th17 cells, positively correlated with thyroid-specific autoantibodies and different thyroid dysfunction stages in HT patients, in which we speculate that MIF may also be involved in the pathogenesis and development of thyroid autoimmune responses in patients with HT. Given that current therapeutic investigations revealed that MIF and IL-17 deficiency through genetic deletion or Abs neutralization results in protection or release from several animal models of inflammatory and autoimmune disease [9, 10, 24–27, 36], clinical application still needs further in-depth research. Anyhow better advances of MIF and Th17 cells in the pathogenesis of HT will be helpful for the new target of treatment for patients with HT.

## Figures and Tables

**Table 1:** Characteristics of patients with HT and healthy controls.

	HT	HT-A	HT-B	Healthy control	Normal range
Cases (*n*)	40	22	18	30	—
Gender (F/M)	35/5	19/3	16/2	26/4	—
Age (years)	28.93 ± 8.30	28.91 ± 9.21	28.94 ± 7.30	30.97 ± 6.39	—
TSH (mIU/L)	4.13 (1.82–6.25)^**^	1.94 (0.99–3.06)	6.95 (5.12–16.18)^**^	1.89 (1.15–2.59)	0.28–4.3
FT_3_ (pmol/L)	4.49 ± 1.32^*^	4.97 ± 1.17	3.89 ± 1.27^**^	5.23 ± 0.98	2.8–7.1
FT_4_ (pmol/L)	15.65 ± 3.04^*^	16.58 ± 2.28	14.52 ± 3.50^**^	17.29 ± 2.09	12–22
TPOAb (IU/mL)	200.00 (148.35–299.88)^**^	171.00 (109.53–215.08)^**^	294.75 (199.58–410.85)^**^	14.25 (7.53–24.00)	0–34
TgAb (IU/mL)	489.75 (300.70–774.70)^**^	335.15 (258.85–485.48)^**^	717.55 (551.53–1055.58)^**^	51.00 (19.75–84.25)	0–115
MUI (*μ*g/L)	169.40	168.45	170.30	163.35	—

Data are shown as mean ± SD or median (25th–75th percentile) according to the distribution. HT-A, HT patient with normal thyroid function; HT-B, HT patient with subclinical or overt hypothyroidism; M, male; F, female; MUI, median of urine iodine. The *P* values represent different groups compared with healthy control (HC), ^*^P < 0.05, ^**^P < 0.01.

**Table 2:** Primers for real-time quantitative RT-PCR.

Primer	Sequence (5′ to 3′)
MIF	Forward: 5′-ACCAGCTCATGGCCTTCG-3′
	Reverse: 5′-CTTGCTGTAGGAGCGGTT-3′

IL-17A	Forward: 5′-TGTCCACCATGTGGCCTAAGAG-3′
	Reverse: 5′-GTCCGAAATGAGGCTGTCTTTGA-3′

*β*-actin	Forward: 5′-AGTTGCGTTACACCCTTTCTTG-3′
	Reverse: 5′-TCACCTTCACCGTTCCAGTTT-3′

## Abbreviations

MIF: Macrophage migration inhibitory factor
HT: Hashimoto's thyroiditis
AITD: Autoimmune thyroid disease
Th17: T helper 17
IL-17A: Interleukin 17A
PBMC: Peripheral blood mononuclear cell
TPOAb: Thyroid peroxidase antibody
TgAb: Thyroglobulin antibody
TSH: Thyrotropin
FT_3_: Free triiodothyronine
FT_4_: Free thyroxine
MUI: Median of urine iodine
ELISA: Enzyme-linked immunosorbent assay
ECLIA: Electrochemiluminescence immunoassay analyzer.
